# Repeated microdissection testicular sperm extraction in patients with non-obstructive azoospermia: Outcome and predictive factors

**DOI:** 10.1080/2090598X.2022.2028066

**Published:** 2022-01-24

**Authors:** Ibrahim Fathi Ghalayini, Rami Alazab, Omar Halalsheh, Alia H. Al-Mohtaseb, Mohammed A. Al-Ghazo

**Affiliations:** a Urology Division, King Abdullah University Hospital/Jordan University of Science and Technology, Amman, Jordan; b Pathology and Laboratory Department, King Abdullah University Hospital/Jordan University of Science and Technology, Amman, Jordan

**Keywords:** Azoospermia, repeated tese, testicular sperm, TESE

## Abstract

**Objective:**

To assess the feasibility of repeated sperm recovery in patients with non-obstructive azoospermia (NOA), as little is known about the extraction rate in repeated microdissection testicular sperm extraction (microTESE) in these patients.

**Patients and Methods:**

A total of 134 men with NOA had their first sperm recovery between January 2013 and February 2020. Repeated microTESE had been done mostly for patients with a successful initial retrieval.

**Results:**

In the 323 procedures performed on the 134 men with NOA, sperm could be retrieved in 236 procedures (73.1%). A total of 88, 61 and 40 men underwent two, three and four sperm retrievals, respectively. In these cycles, sperm could be extracted in 65 (73.9%), 53 (86.9%) and 37 (92.5%) men, respectively. During the first microTESE procedure, sperm could be extracted in 81 (60.4%) men with NOA. In all, the success rate was significantly different between subgroups, showing highest rate in hypospermatogenesis cases (95.6%), followed by maturation arrest (58.5%), and Sertoli cell-only syndrome (56.0%). However, this difference was not significant at the third and fourth repeated microTESE. The FSH levels and testicular volume were among the noticeable factors affecting success of sperm retrieval. The duration between the first and second biopsies significantly increased the success rate by a factor of 1.3-fold/month; however, afterwards, the duration did not play any role in the success of microTESE. The success of previous trial significantly increased the probability of success by 10.1-fold in the second trial, 5.6-fold in the third trial, and 16.5 folds in the fourth.

**Conclusion:**

Repeated MD -TESE ensures a high sperm recovery rate in patients with NOA. These data also show that when no spermatozoa can be obtained after thawing cryopreserved testicular sperm for ICSI in NOA patients, a repeat microTESE procedure can be planned.

**Abbreviations:**

ICSI: intracytoplasmic sperm injection; IVF: in vitro fertilisation; MA: maturation arrest; (N)OA: (non-)obstructive azoospermia; OR: odds ratio; SCOS, Sertoli cell-only syndrome; SRR: spermatozoa retrieval rate; (micro)TESE: (microdissection) testicular sperm extraction

## Introduction

Non-obstructive azoospermia (NOA) refers to absence of spermatozoa in semen analysis due to minimal or no production of fully developed spermatozoa in the testicles. Aetiologies for testicular failure include genetic disorders such as sexual chromosomal abnormalities, translocations and microdeletions of the Y chromosome, cryptorchidism, testicular torsion, radiation, and toxins [1,2]. Approximately 1% of all men and 10% of infertile men are affected by testicular failure as a result of NOA [3]. Testicular spermatozoa can be retrieved in some men with NOA despite the absence of ejaculated spermatozoa in their semen, because of the existence of isolated foci of active spermatogenesis. Testicular spermatozoa can be retrieved successfully by the testicular sperm extraction (TESE) procedure and used for intracytoplasmic sperm injection (ICSI) in cases of NOA [2]. Such cases used to be treated with conventional TESE, including multiple biopsy samples of the testis. At present, in many centres this treatment has been replaced by microdissection TESE (microTESE).

Direct vision with the operating microscope in microTESE is of great advantage as larger, more opaque, whitish tubules, presumably containing more germ cells with active spermatogenesis, can be identified. This procedure is currently the best method for the identification of sperm, resulting in a high spermatozoa retrieval rate (SRR) and minimal postoperative complications. Histological findings are important in any comparison, as a relationship between the SRR and testicular histopathology has been reported in the context of conventional TESE [3,4].

In OA, sperm can be retrieved in almost 100% of the cases [5]. However, in NOA the possibility of finding sperm is only ~50% by conventional TESE [4,5]. Furthermore, if testicular sperm is found in patients with NOA, the pregnancy rate after one ICSI cycle is low [6], and repeated ICSI cycles with repeated testicular biopsies are therefore often needed. Only a few studies, based on a limited number of cases, have examined the possibility of finding sperm in repeated conventional testicular biopsies in patients with azoospermia [7–10] and little research performed for repeated microTESE. Therefore, in the present study, we examined whether consecutive microTESE are successful in a large series of patients with azoospermia, with the hope our study would add information to support the previous reports. Therefore, we examined whether repeated microTESE procedures are successful in patients with NOA. Diagnostic histological biopsy specimens were reviewed in all cases. We relate the positive sperm recovery to certain variables: FSH and LH concentrations, testicular volume, and testicular histology, which are all clinically relevant for patients with NOA.

## Patients and methods

In this retrospective case series, we included patients who had their first microTESE procedure for ICSI, between January 2013 and February 2020. All patients were diagnosed with NOA based on a complete history, physical examination, endocrine profile, and chromosomal analysis before being scheduled for microTESE with sperm freezing. Those with abnormal karyotyping were excluded from analysis. All patients underwent ejaculated semen examination at least three times before surgery. Patients also underwent careful evaluation by urologists concerning the duration of sterility, medical history, sexual function, and results of a gynaecological evaluation of the spouse. Ultrasonography was performed to measure testicular volume and to determine the status of the epididymis and testis. Serum FSH and LH were measured by immunoradiometric assay, while testosterone was measured by radioimmunoassay. Patients included in the study were allocated, according to the waiting list on the basis of the general operative theatre plan, after informed consent including explanations about results in the literature and invasiveness of the procedure. Every operated testicle was classified according to the following variables: (i) testicular volume, categorised according to volume (≤8, 9–12, and ≥13 mL, i.e. normal); (ii) FSH concentration, categorised into two groups according to multiples of the normal range (N) (N and 2 N;1–24 mIU/mL and >3 N; >24 mIU/mL); (iii) testicular histology based on the most advanced pattern of spermatogenesis present such as hypospermatogenesis, maturation arrest (MA) and Sertoli cell-only syndrome (SCOS). Institutional Review Board approval was obtained from the Ethics Committee at the Jordan University of Science and Technology.

### Surgical approach

MicroTESE was performed under general anaesthesia according to the procedure reported previously [11,12]. After the tunica albuginea was opened widely along the antimesenteric border, direct examination of the testicular parenchyma was performed under the operating microscope at ×10–24, according to the protocol described by Schlegel [11]. An attempt was made to identify individual seminiferous tubules that were larger, opaquer, and whiter than other tubules in the testicular parenchyma, which were considered to contain spermatozoa. The procedure was terminated when sperm were retrieved, or further biopsy was thought likely to jeopardise the blood supply of the testis. If all tubules were seen to have an identical morphological appearance, at least three samples (upper, middle, and lower) that were equivalent to those from multiple TESE were obtained. The procedure was terminated when a sufficient volume of spermatozoa had been retrieved for ICSI. At the same time of testicular intervention in both procedures, a small tissue specimen was placed in Bouin’s solution and sent for histopathological examination. The testicular cell suspension was frozen for later use, if at least one, preferably motile, sperm was observed after diagnostic retrieval or if, after injection of the mature oocytes at the day of biopsy retrieval, sufficient remaining spermatozoa were supposed to be available for a next ICSI treatment.

### Statistical analysis

The results were statistically evaluated using Mann–Whitney and Pearson’s chi-square tests for comparison of the baseline continuous and categorical variables, respectively. A binary logistic regression was used to assess the adjusted odds ratios (ORs) for several factors affecting sperm retrieval success rate. A *P* < 0.05 was considered statistically significant. All data were analysed using the Statistical Package for the Social Sciences program (SPSS®) for Windows 16.0, SPSS Inc., Chicago, IL, USA).

## Results

### Patient background

NOA was diagnosed in 134 men who underwent microTESE. The microTESE procedure had been performed as a primary procedure without a previous minimally invasive method and by the same long-standing experienced surgeon. Preoperative patient characteristics including endocrine data and histopathological diagnosis are summarised in Table 1.

**Table 1.** Baseline data of 134 patients with repeated microTESE groups.

Variable	Value
Age, years, mean (SD)	34.9 (8.4)
FSH level, IU/L, mean (SD)	19.1 (12.4)
FSH (IU/L), *n* (%)	
≤12	48 (35.8)
13–24	44 (32.8)
>24	42 (31.3)
Testicular volume, mL, mean (SD)	11.9 (4.1)
Testicular volume (mL), *n* (%)	
≤8	28 (20.9)
9–12	56 (41.8)
≥13	50 (37.3)
Patients with varicocele, *n* (%)	22 (16.4)
Patients after orchidopexy, *n* (%)	7 (5.2)
Histopathological diagnosis, *n* (%)	
Hypospermatogenesis	58 (43.3)
MA	22 (16.4)
SCOS	54 (40.3)

In the 323 procedures performed on the 134 men with NOA, sperm could be retrieved in 236 procedures (73.1%). During the first microTESE procedure, sperm was extracted in 81 men (60.4%). In repeated procedures, sperm could be extracted in 155 of the 189 microTESE procedures (82%). A total of 88, 61 and 40 patients underwent two, three and four sperm retrievals, respectively. The extraction rate in the consecutive cycles among patients with NOA is summarised in Table 2. Although it is our policy not to repeat a TESE in case of previous failed surgery, two patients who had a first successful and a second unsuccessful TESE chose to undergo a third one. In one case, the third biopsy was successful. The same testis had been used for the repeated procedure, while the contralateral testis opened in case of failure of the previous one or if adequate sperm could not be achieved.

**Table 2.** Consecutive sperm retrieval in patients with NOA.

Rank	*N*	Successful sperm retrieval, *n* (%)
Total	323	236 (73.1)
First	134	81 (60.4)
Second	88	65 (73.9)
Third	61	53 (86.9)
Fourth	40	37 (92.5)

### Role of histological diagnosis

During the first microTESE procedure, sperm could be extracted in 17 of the 54 (31.5%) patients with a germ cell aplasia (SCOS), in nine of the 22 (40.9%) patients with a maturation arrest, and in 55 of the 58 (94.8%) patients with hypospermatogenesis The extraction rate in the consecutive cycles among the different subgroups of patients with NOA is summarised in Table 3. The influence of histological diagnosis on the success rate of sperm retrieval was evaluated. The success increases by 40-fold in patients with hypospermatogenesis when compared to SCOS in the first trial (OR 39.9, *P* < 0.001, Table 4). We obtained spermatozoa in 94.8% of those with the histological diagnosis of hypospermatogenesis compared to 40.9% and 31.5% of men with maturation arrest and SCOS, respectively. At the second trial the success increases by 25-fold in patients with hypospermatogenesis when compared to SCOS (OR 24.5, *P* < 0.001, Table 5).

**Table 3.** Consecutive sperm retrieval in the subgroups of patients with NOA.

Rank	Hypospermatogenesis	MA	SCOS	*P*
	Successful sperm recovery, *n* (%)	Successful sperm recovery, *n* (%)	Successful sperm recovery, *n* (%)	
First	55 (94.8)	9 (40.9%)	17 (31.5%)	<0.001*
Second	36 (97.3)	7 (50.0%)	22 (59.5%)	<0.001*
Third	22 (91.7)	9 (81.8%)	22 (84.6%)	0.7
Fourth	17 (100)	6 (100%)	17 (100%)	0.1
Total	130/136 (95.6)	31/53 (58.5%)	75/134 (56.0%)	0.001*

*Chi-square test showed significant difference in successful recovery between subgroups.

**Table 4.** Factors affecting success of sperm retrieval at first trial.

Variable	OR (95% CI)	P
Age	1.006 (0.965–1.049)	0.8
FSH (IU/L)	0.951 (0.923–0.981)	0.001*
Testicular volume	1.355 (1.179–1.557)	<0.001*
Histopathological diagnosis		
Hypospermatogenesis	39.902 (10.916–145.856)	<0.001*
MA	1.507(0.540–4.203)	0.4
… SCOS	1	

*Statistically significant.

**Table 5.** Factors affecting success of sperm retrieval at second trial.

Variable	OR (95% CI)	*P*
Success of first trial	10.095 (3.405–29.935)	<0.001*
Duration between first and second biopsies	1.314 (1.062–1.627)	0.01*
Age	0.980 (0.926–1.038)	0.5
FSH (IU/L)	0.973 (0.938–1.009)	0.1
Testicular volume	1.244 (1.048–1.477)	0.01*
Histopathological diagnosis		
Hypospermatogenesis	24.545 (3.028–198.960)	<0.001*
MA	0.682 (0.198–2.347)	0.5
… SCOS	1	

*Statistically significant.

### Role of FSH concentration

Elevated FSH levels showed significant rates of sperm retrieval failure (OR 0.95, *P* = 0.001, Table 4). Lower FSH concentration was significantly correlated with the success rate of sperm retrieval in the first trial (*P* = 0.001). However, no significant role was shown in the second, third or fourth procedures. No hormonal stimulation was used before the procedures and the baseline tests were not repeated before each procedure.

### Role of interval duration between procedures

The success of consecutive trials increased when the preceding one was successful (Tables 4–Table 6). An increase in the duration was only significant between the first and second trial (OR 1.3, *P* = 0.01, Table 5). However, after the second and third trial the duration had no significant effect on the success rate. So the interval duration was only significant between first and second procedure. In all, 68 of the trials out of 323 (21%) were performed with an interval just <6 months.

**Table 6.** Factors affecting success of sperm retrieval at third trial.

Variable	OR (95% CI)	*P*
Success of first trial	4.889 (1.027–23.275)	0.05*
Success of second trial	5.625 (1.162–27.222)	0.03*
Duration between second and third biopsies	0.980 (0.802–1.198)	0.8
Age	1.058 (0.937–1.194)	0.4
FSH (IU/L)	1.000 (0.938–1.065)	1
Testicular volume	1.038 (0.830–1.299)	0.7
Histopathological diagnosis		
Hypospermatogenesis	2.000 (0.331–12.067)	0.5
MA	0.818 (0.127–5.288)	0.8
… SCOS	1	

*Statistically significant.

### Role of testicular volume

Higher testicular volume was significantly correlated with success rate of sperm retrieval in the first trial (OR 1.355; *P* < 0.001, Table 4) and second trial (OR 1.244; *P* = 0.01, Table 5) but not in the third or fourth trial (Tables 6 and 7).

**Table 7.** Factors affecting success of sperm retrieval at fourth trial.

Variable	OR (95% CI)	*P*
Success of first trial		N/A
Success of second trial	4.125 (0.302–56.385)	0.3
Success of third	16.500 (1.207–225.541)	0.04*
Duration between third and fourth biopsies	0.972 (0.679–1.392)	0.9
Age	1.038 (0.858–1.257)	0.7
FSH (IU/L)	0.882 (0.770–1.009)	0.07
Testicular volume	1.371 (0.867–2.169)	0.2
Histopathological diagnosis		
Hypospermatogenesis		N/A
MA		N/A
… SCOS	1	

*Statistically significant.

## Discussion

Although repeated procedures are often required to obtain adequate sperm samples, information regarding the outcome of repetitive microTESE procedures is scarce. Cryopreservation of remaining testicular tissue is a valid option to avoid repeated surgery. However, cryopreservation is not always feasible in NOA, and no sperm will be found after thawing in ~20% of patients; a repeat TESE procedure therefore needs to be performed on the day of oocyte retrieval [10]. Some couples may also need a repeat procedure to achieve a second pregnancy.

Because the quantity of testicular tissue is limited and some authors have cautioned for possible testicular damage after TESE [13], the true prognostic value of repetitive TESE is of paramount importance to adequately counsel patients. MicroTESE facilitates the removal of smaller amounts of testicular tissue, which becomes crucial in the presence of testicular atrophy. In addition, the identification of avascular regions for the opening of the tunica albuginea could minimise the chances of vascular injury. Multiple biopsy samples from different regions of the testis may increase the possibility of detecting spermatozoa with conventional TESE.

Talas et al. [14] evaluated the outcome of repetitive microTESE attempts among 68 patients with NOA. The first microTESE yielded mature sperm for ICSI in 44 (64%) of the patients and failed in the remaining 24 (36%). Following their first trial, 24 patients decided to undergo a second microTESE. Of these 24 patients, no spermatozoa were obtained in five, while achieved in 19. In these 24 cases, microTESE was successively repeated for two (24), three (four) and four (one) times. The second attempt yielded mature sperm in three of five patients from the sperm-negative group and 16/19 patients from the sperm positive group. At the third and fourth trials, sperm retrieval was achieved in four of four and one of one patients, respectively. Distribution of main testicular histology included SCOS (16%), MA (22%), hypospermatogenesis (21%), and focal spermatogenesis (41%). Overall, in repetitive mTESE, 24/29 (82%) of the attempts were finally successful. Similar to our findings with a larger number of trials in which the authors concluded that in patients with NOA, microTESE may safely be repeated one or more times to increase the SRR, as well as to increase the chance of retrieving fresh spermatozoa to enable ICSI.

Westlander et al. [7] examined the feasibility of repeating testicular sperm aspiration in 34 men with NOA. They found that, in one patient, repeating the procedure up to the sixth attempt was feasible. However, their definition of NOA was unclear as no histology was available and may have included a substantial proportion of patients with normal spermatogenesis or hypospermatogenesis. In contrast, we included the histology for each patient in the present study and found that repeating microTESE was feasible. Friedler et al. [8] examined repeated TESE in 22 patients with NOA defined according to histology. Repeating the procedure up to the four times was justified. These findings were corroborated by Kamal et al. [9] in a study of 41 patients with NOA. Repeated TESE trials were successful in 91.5% of patients if sperm was recovered during the first procedure. Xu et al. [15] reported that salvage microTESE is of clinical value in men with NOA with failed TESE attempts. Our findings that were based on a large number of patients who had repeated microTESE, with a well-defined azoospermia, are in agreement with the previous reported studies.

In a large study performed by Vernaeve et al. [10] on 628 men with NOA, with up to sixth trials of TESE concluded that repeated TESE ensures a high sperm recovery rate in patients with NOA. Our findings are in line with this study using microTESE.

To determine the predictive value of a previous testicular biopsy on the chance of sperm retrieval in the next TESE procedure, we analysed the outcome of past sperm collection procedures and histopathology diagnoses of patients with NOA. Repeated TESE ensured a high recovery rate (92.5%) when the first recovery procedure had been successful and when hypospermatogenesis was diagnosed (95.6%). Kavoussi et al. [16] concluded in their study that men with NOA who underwent microTESE with a hypospermatogenesis testicular histopathology had better outcomes including rates of sperm retrieval, as well as downstream outcomes specifically clinical pregnancy, live birth, and having enough sperm retrieved for more than one *in vitro* fertilisation (IVF)/ICSI cycle, over those with histopathological patterns of MA and SCOS. Haimov-Kochman et al. [17] reported when no spermatozoa were found on the first attempt, a repeat TESE procedure was successful in one-third of the patients.

Clinically, testicular volume is correlated with spermatogenesis. Some authors reported testicular volume had poor predictive value for successful TESE; however, because topographical variations in testicular pathology, independence of testicular volume, can occur [18]. Indeed, it had been reported that there is no statistically significant difference in testicular volume between patients with retrievable spermatozoa and those without [18,19]. Furthermore, no lower limit of testicular volume for the absence of spermatozoa has been identified. Spermatozoa are often retrieved from testes with volumes <5 mL by microTESE. Thus, small testicular volume itself does not preclude successful microTESE [18,20]. We found a positive relation between the SRR and testis volume as well [21–23]. In our present study, higher testicular volume was significantly correlated with success rate of sperm retrieval in the first trial (OR 1.355, *P* < 0.001) and second trial (OR 1.244, *P* = 0.01) but not in the third or fourth trial. Turunc et al. [21] reported a significantly lower SRR (20.8%) in the patients who had testis volumes of ≤5 mL. Therefore, it can be suggested that patients with NOA whose testis volumes are lower should be informed about the low SRR with TESE. Another important issue in TESE is the amount of removed testicular tissue in the operation. Large amounts of removed testicular tissue may cause testicular insufficiency with a decrease in testosterone levels, especially in hypoplastic/atrophic testicles. Many authors reported that the amount of removed testicular tissue in microTESE was significantly lower than with the conventional method [11,24]. We could not measure the amount of testicular tissue removed in patients during the TESE operation. This missing information is a possible limitation of our present study.

Ramasamy et al. [25] reported a lower SRR in the group of patients with FSH levels of <15 IU/mL, while high serum FSH levels in men with NOA did not affect the success of microTESE. In our present study, a lower FSH concentration was significantly correlated with success rate of sperm retrieval in the first trial (*P* = 0.001). However, no significant role was shown in the second, third or fourth procedures. Turunc et al. [21] reported no significant relation between FSH levels and the SRR. In our previous study [23], increase in FSH levels showed significant failure of sperm retrieval in general, which was more significant in conventional TESE. Although previous studies revealed a negative correlation between increased FSH levels and the SRR, recent studies showed no significant relation between FSH levels and the SRR [21]. Consistent with the literature, a significant relationship between FSH levels and the SRR was detected in our present study.

As time is required for recovery of the limited sperm production that is present in men with NOA, ~6 months should be allowed after microTESE before considering repeat microTESE procedures if additional attempts are required [13,26,27]. Repeat TESE procedures were far more likely to retrieve spermatozoa if the second TESE attempt was performed >6 months after the initial TESE procedure. Transient adverse physiological effects are common in the testis for up to 6 months after TESE [13]. We have only a few cases who decided to repeat the procedure between 3 to 6 months. The success of consecutive trials increased when the preceding one was successful. The increase in the duration was only significant between the first and second trial (OR 1.3, *P* = 0. 01, Table 5). However, after the second, the duration had no significant effect on the success rate.

Our present study included a small number of trials performed between 3 to 6 months intervals for logistic reasons, which may be a limitation of this study. This is retrospective study, and some patients had a trial by different surgeons outside our centre. Few patients came back with foci of spermatogenesis in histology reports and insisted to repeat the trial with assumption of an IVF laboratory technical error. Some patients asked to repeat the trial after completing supplements recommended by pharmacological companies. A reasonable number of patients live in another country for work with limited holidays to stay.

Being a retrospective study is a limitation of this study and we are currently working on prospective research. Baseline tests were not repeated before each procedure. This may be a minor limitation in our study.

We may conclude that repeated microTESE ensures a high recovery rate in patients with NOA, especially when a first recovery procedure has been successful. Our data also show that when no spermatozoa can be obtained after thawing cryopreserved testicular sperm for ICSI in patients with NOA, a repeat TESE procedure can be scheduled. Performing microdissection is still the most effective treatment alternative in terms of a high SRR and fewer complications. There was a relation between the SRR and testicular volume and FSH levels. MicroTESE appears to be endorsed especially in cases of atrophied testicles, high FSH concentration, or when SCOS with high FSH concentration can be predicted on the basis of the preoperative prognostic data.
